# Nutrient Scarcity in a New Defined Medium Reveals Metabolic Resistance to Antibiotics in the Fish Pathogen *Piscirickettsia salmonis*

**DOI:** 10.3389/fmicb.2021.734239

**Published:** 2021-10-11

**Authors:** Javiera Ortiz-Severín, Camila J. Stuardo, Natalia E. Jiménez, Ricardo Palma, María P. Cortés, Jonathan Maldonado, Alejandro Maass, Verónica Cambiazo

**Affiliations:** ^1^Laboratorio de Bioinformática y Expresión Génica, Instituto de Nutrición y Tecnología de los Alimentos, Universidad de Chile, Santiago, Chile; ^2^Fondap Center for Genome Regulation (Fondap 15200002), Universidad de Chile, Santiago, Chile; ^3^Centro de Modelamiento Matemático (AFB170001), Departamento de Ingeniería Matemática, Facultad de Ciencias Físicas y Matemáticas, Universidad de Chile and UMI-CNRS 2807, Santiago, Chile

**Keywords:** *P. salmonis*, fish pathogen, defined medium, nutrient scarcity, antibiotic resistance, metabolic resistance

## Abstract

Extensive use of antibiotics has been the primary treatment for the Salmonid Rickettsial Septicemia, a salmonid disease caused by the bacterium *Piscirickettsia salmonis*. Occurrence of antibiotic resistance has been explored in various *P. salmonis* isolates using different assays; however, *P. salmonis* is a nutritionally demanding intracellular facultative pathogen; thus, assessing its antibiotic susceptibility with standardized and validated protocols is essential. In this work, we studied the pathogen response to antibiotics using a genomic, a transcriptomic, and a phenotypic approach. A new defined medium (CMMAB) was developed based on a metabolic model of *P. salmonis*. CMMAB was formulated to increase bacterial growth in nutrient-limited conditions and to be suitable for performing antibiotic susceptibility tests. Antibiotic resistance was evaluated based on a comprehensive search of antibiotic resistance genes (ARGs) from *P. salmonis* genomes. Minimum inhibitory concentration assays were conducted to test the pathogen susceptibility to antibiotics from drug categories with predicted ARGs. In all tested *P. salmonis* strains, resistance to erythromycin, ampicillin, penicillin G, streptomycin, spectinomycin, polymyxin B, ceftazidime, and trimethoprim was medium-dependent, showing resistance to higher antibiotic concentrations in the CMMAB medium. The mechanism for antibiotic resistance to ampicillin in the defined medium was further explored and was proven to be associated to a decrease in the bacterial central metabolism, including the TCA cycle, the pentose-phosphate pathway, energy production, and nucleotide metabolism, and it was not associated with decreased growth rate of the bacterium or with the expression of any predicted ARG. Our results suggest that nutrient scarcity plays a role in the bacterial antibiotic resistance, protecting against the detrimental effects of antibiotics, and thus, we propose that *P. salmonis* exhibits a metabolic resistance to ampicillin when growing in a nutrient-limited medium.

## Introduction

Over the past few decades, the farmed salmon industry has grown substantially, becoming the largest single fish commodity by value and accounting for approximately 60% of the salmon produced worldwide (Food and Agriculture Organization of the United Nations FAO, 2018). However, the growth of salmon production has encountered important sanitary and environmental concerns, as the aquaculture sector is vulnerable to exotic, endemic, and emerging epizootics (Food and Agriculture Organization of the United Nations FAO, 2018; Jia et al., 2020). Salmonid Rickettsial Septicemia (SRS) is a bacterial disease first reported in 1980 in southern Chile (Bravo and Campos, 1989; Cvitanich et al., 1991); since then, it has been described in different salmonid and non-salmonid fish in other countries such as Australia and New Zealand, United Kingdom, Norway, and Canada (Rozas and Enriquez, 2014). In Chile, epizootics events of SRS have occurred in all farmed salmonid species, accounting for 50–97% of infectious disease mortality during the seawater growing phase (Mardones et al., 2018) and causing up to US$ 700 million in losses in the Chilean salmon industry every year (Avendaño-Herrera, 2018; Mardones et al., 2018).

The etiological agent of the SRS disease is the Gram-negative γ-Proteobacteria *Piscirickettsia salmonis* (Cvitanich et al., 1991; Fryer et al., 1992), a non-motile, aerobic, facultative intracellular pathogen that replicates inside cytoplasmic vacuoles, survives for long periods of time in seawater, and can be directly transmitted from the surrounding water to the fish (Lannan and Fryer, 1994; Smith et al., 1999; Jones et al., 2020). So far, vaccines have not proven to be effective toward SRS, thus forcing to rely on antimicrobials as treatment. In Chile, intensive usage of antimicrobials has taken place at least since 2007, with florfenicol and oxytetracycline being the most used antibiotics, which have proven to be effective against *P. salmonis* infections and accounted for nearly 98% of the antibiotics administered by this industry (SERNAPESCA, 2020). The excessive use of antibiotics in the Chilean salmon industry has led to several studies exploring the effects on the selection of resistant bacteria in the marine environment and in the farmed salmonids. Both antibiotic-resistant marine microorganisms and antibiotic resistance genes (ARGs) have been identified in water and sediments near salmon farms (Miranda and Rojas, 2007; Tamminen et al., 2011; Shah et al., 2014) and in salmon gut microbiota (Higuera-Llantén et al., 2018). Also, a higher proportion of quinolone-resistant *Escherichia coli* has been detected in the urinary tract of patients from a region with intensive aquaculture, suggesting that antibiotic resistance might have been transmitted from the farm environments to human pathogens (Tomova et al., 2015). Nevertheless, to this date, only one strain of *P. salmonis* has been described as resistant to the antibiotics used in aquaculture, a strain that harbors a multidrug resistance plasmid conferring resistance to oxytetracycline, chloramphenicol, streptomycin, and sulfamethoxazole/trimethoprim (Saavedra et al., 2018).

Although some reports have explored the potential antibiotic resistance of as much as 292 *P. salmonis* isolates (Yánez et al., 2013; Henríquez et al., 2016; Mancilla, 2018), all isolates were susceptible to the antibiotics administered for SRS treatment, at the recommended concentrations used in medicated feed (San Martín et al., 2019). Point mutations in *gyrA* (Henríquez et al., 2014), single-nucleotide polymorphisms in putative oxytetracycline and florfenicol resistance genes (Figueroa et al., 2019), the expression of tetracycline and florfenicol putative resistance genes along with multidrug pumps (Cartes et al., 2017), and an efflux pump modulated by florfenicol (Sandoval et al., 2016) have been described in different *P. salmonis* strains. However, all reports informed discrete minimum inhibitory concentration (MIC) values associated with the putative resistant phenotype. In accordance with the recommendations of the European Committee on Antimicrobial Susceptibility Testing (EUCAST), the definition of a “susceptible” or “resistant” microorganism should be related to the likelihood of therapeutic success or failure using a standard dosing regimen of the agent (EUCAST, 2019). Besides that, additional aspects need to be considered, among them, the inherent variability of MIC measurements that can be attributed to the difficulty of growing a particular fastidious organism (such as *P. salmonis*) or to unexpected effects of the combination of the organism, the culture media, and the antimicrobial agent (EUCAST, 2019).

According to recommendations of the Clinical Laboratory Standard Institute (CLSI), antimicrobial susceptibility testing to determine *P. salmonis* MIC values are performed in Austral-SRS (Miller et al., 2014), a nutrient-rich and undefined medium. However, several reports have brought into question the *in vivo* relevance of *in vitro* susceptibility testing in enriched culture media. Considering that the *in vivo* environment of bacterial pathogens is frequently regarded as nutrient-limited, nutrient-rich culture conditions may have a poor predictive value of antibiotics effectivity (Ersoy et al., 2017; Farha et al., 2018; Sanders et al., 2018). In addition, the inhibitory effects of antibiotics could be misleading as growth media composition can significantly alter bacterial metabolism (Pethe et al., 2010; Hicks et al., 2018). Thus, these studies highlight the need for a defined minimal medium that provides a more consistent nutritional setting for determining the susceptibility of *P. salmonis* to antibiotics. Furthermore, several reports have illustrated that the metabolic state of bacteria significantly affects their susceptibility to the antibiotics (Stokes et al., 2019). As described for *E. coli* (Lopatkin et al., 2019), in a metabolic coupled scenario, the bacterial growth rate positively correlates with its metabolism (i.e., an increase in metabolism generates energy that in turn increases bacterial growth), and both growth and metabolism are nutrient-limited. However, when growth is nutrient-limited in the presence of an excess of energy, nutrient availability is correlated with growth but not with the metabolism and metabolic uncoupling takes place (Erickson et al., 2017; Lopatkin et al., 2019). In a metabolic uncoupled scenario, the bacterial metabolic state more accurately predicts the antibiotic lethality. Similarly, increasing the basal respiration rate of *E. coli* by genetically uncoupling ATP synthesis from electron transport improves bactericidal antibiotic efficacy (Lobritz et al., 2015). The modulation of antibiotic resistance by bacterial metabolism is also supported by reports on phenotypic resistance, a transient and reversible state of reduced susceptibility to antibiotics that is linked to the metabolic state of bacteria (Martínez and Rojo, 2011).

Since knowing the minimal metabolic requirements for *P. salmonis* growth is an important first step to decipher the mechanisms of *P. salmonis* susceptibility to antibiotics, here, we present a new culture medium with defined composition (CMMAB) with adjusted divalent cations, in compliance with CLSI standards (Miller et al., 2014; CLSI National Committee for Clinical Laboratory Standards, 2015) that supports growth of four *P. salmonis* strains. Both the CMMAB and the Austral-SRS media were utilized to conduct susceptibility testing of *P. salmonis* against a series of antibiotics for which a resistant phenotype was expected based on *in silico* prediction of *P. salmonis*-encoded ARGs. For macrolides, beta-lactams and aminoglycosides, an increase in bacterial resistance was detected in the CMMAB compared to the Austral-SRS medium. Ampicillin inhibited bacterial growth in Austral-SRS media, along with a decrease in doubling time, and an increase in carrying capacity. Phenotypic and transcriptomic data revealed that the bacterial metabolic state plays an important role in the resistance of *P. salmonis* to ampicillin, as bacterial energy production metabolism, TCA, pentose phosphate pathways, and nucleotide metabolism were downregulated in the CMMAB media, both with and without the antibiotic. Thus, the CMMAB medium can be a useful tool for identifying essential metabolic functions of *P. salmonis*, improving approaches for antimicrobial testing and drug design against this fish pathogen.

## Results

**FIGURE 1:**
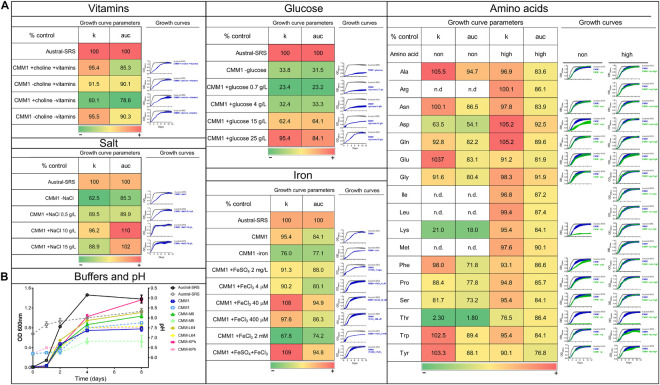
Evaluation of nutrient composition and requirements for optimal growth of *P. salmonis* LF-89. Growth curves and growth curve parameters of *P. salmonis* LF-89 in the nutrient-rich medium Austral-SRS and the defined medium CMM1 with nutrient modifications. **(A)** The carrying capacity parameter (k) and the area under the curve of the fitted logistic equation for the OD_600_ measurements over time (auc), with its corresponding growth curve, for the bacterial growth in Austral-SRS medium as control, CMM1 defined medium or CMM1 with modifications in the vitamins, salt, glucose, iron, and amino acid constituents. In the growth curve graphs, the control media (Austral-SRS) is shown in gray, and CMM1 media and its modifications are in blue. Amino acid changes (in green) were set in two concentrations: 0 g/L (shown in the column “non”) and at a higher concentration that the one used in CMM1 composition (column “high”). Essential amino acids required for bacterial growth were not tested (indicated with n.d.). Growth parameters are presented as percent of the control (Austral-SRS), higher values are shown in red, intermediate values are in yellow, and lower values are in green. **(B)** Growth curves of the bacterial growth (solid lines) and the culture media pH (dotted lines) using different buffers in the media. Specific composition of media is described in Supplementary Table 1.

*In silico* analysis of *P. salmonis* LF-89 nutrient requirements by Cortés et al. (2017) suggested that there is a trade-off between the need for carbon and nitrogen sources for bacterial growth, and accordingly, the highest growth rate was obtained when a mixture of glucose and amino acids were used. Consequently, we sought to obtain the best combination of nutrients to increase bacterial growth. Based on the composition of the defined medium (called here as CMM1), different concentrations of vitamins, iron, glucose, NaCl, and amino acid mixtures were tested and their effect on *P. salmonis* growth was quantified and compared (Figure 1). For this, the area under the curve (auc) and the carrying capacity (k) parameters were selected. The first parameter represents the overall growth of the bacterium in a selected condition, including its replication rate, the highest growth achieved, and the time it took to achieve it; the second parameter represents a measure of bacterial load, which relates to the nutrient availability and the bacterial capacity to use it.

**FIGURE 2:**
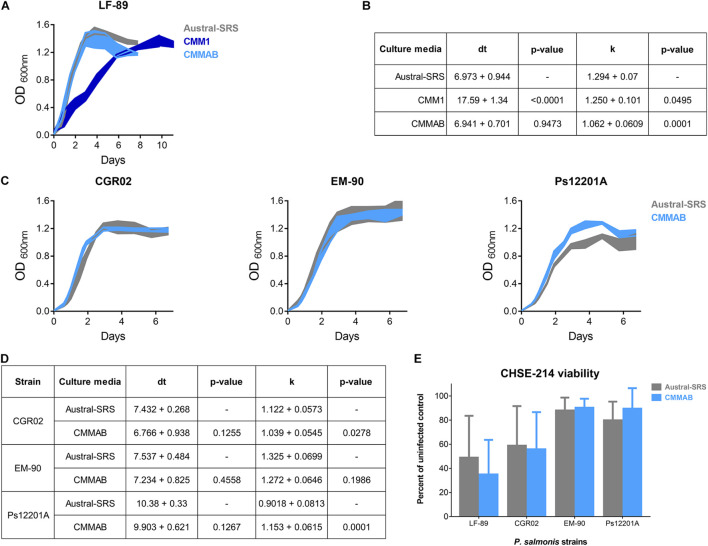
Bacterial infection and growth comparison of different *P. salmonis* strains in nutrient-rich and in a defined culture media. **(A)** Growth curves of the reference strain LF-89 in Austral-SRS nutrient medium, CMM1 defined medium and the optimized defined medium CMMAB. **(B)** The carrying capacity parameter (k) and the maximum doubling time (dt) were obtained by assessing the unrestrained growth of *P. salmonis* LF-89 in the three media. A *t*-test was performed to compare the growth curve parameters between the bacteria grown in the CMM1 or in the CMMAB media with the control Austral-SRS medium. **(C)** Growth curves of *P. salmonis* strains CGR02, EM-90, and Ps12201A in the control nutrient media and the optimized defined media (Austral-SRS and CMMAB, respectively). **(D)** Growth curve parameters k and dt of CGR02, EM-90, and Ps12201A strains in Austral-SRS and CMMAB media. A *t*-test was performed to compare the growth curve parameters between the bacteria grown in CMMAB media and the control Austral-SRS medium. **(E)** Cell viability MTT assay after infection with *P. salmonis* strains grown in Austral-SRS (gray bars) or in CMMAB (light blue bars). A two-way ANOVA showed no significant differences between the effect on cell viability (presented as percent viability of uninfected controls) caused by bacteria grown in both media.

The defined media described in Cortés et al. (2017) allowed for sub-optimal growth in *P. salmonis*, as growth curve parameters such as generational time, carrying capacity, and overall growth curve dynamics (Figures 1, 2) were significantly different from those obtained in the nutrient-rich media Austral-SRS. Therefore, the medium Austral-SRS was used as a positive control for growth. As shown in Figure 1A, *P. salmonis* growth was directly correlated to the glucose concentration in the media, as well as to NaCl concentration up to 10 g/L. Vitamin availability was not determinant in bacterial growth, even though it is reported that *P. salmonis* does not have complete biosynthesis pathways for them (Cortés et al., 2017). In addition, iron availability was important in bacterial growth, as a decrease in almost 20% in the k parameter was observed in the absence of an iron source in the defined medium. The iron oxidation state was also relevant, addition of Fe^3+^ caused the highest increase in growth, and a combination of Fe^2+^ and Fe^3+^ did result in further increasing the bacterial growth.

The CMM1 media contains a mixture of the 20 amino acids (aa), and 7 of them were predicted to be essential for *P. salmonis* because the bacteria does not possess the complete biosynthesis pathways (Cortés et al., 2017). Consequently, auxotrophy for the remaining 13 aa was tested, including a condition with excess of the aa (i.e., a higher concentration than the one used in CMM1). The excess aa condition was also tested for the essential aa arg, ile, leu, and met because *P. salmonis* is able to use them as a carbon source (Cortés et al., 2017). Comparison of bacterial growth curves showed an increased k parameter value in media without ala, asn, glu, trp, and tyr. As a result, a new minimal medium was created based on the minimal nutrient requirements for *P. salmonis* to reach k and auc values comparable to those attained in the nutrient Austral-SRS medium. This minimal medium contains no vitamins, 10 g/L NaCl, 400 μM Fe^3+^, and a mixture of 14 aa. Since the objective of this work was to obtain a culture medium suitable for performing antibiotic susceptibility tests, some components were included in the minimal medium to (1) decrease the pH variability and (2) adjust divalent cations, in order to follow the CLSI standards. This was accomplished by changing the salts supply and including a buffer. For that, several media composition were tested based on other media described for bacterial cultures (Chamberlain, 1965; Cold Spring Harbor Protocols, 2006; Cortés et al., 2017). The buffer and salts composition that allowed better growth and less pH change over time was the CMM-KPh (Figure 1B), and therefore was incorporated in the minimal medium to create a new defined medium optimized for *P. salmonis* growth and suitable for antimicrobial susceptibility testing, named CMMAB. In addition, vitamins were included in this medium as the bacterium cannot synthesize them and other processes besides growth could be affected by their absence. The complete composition of each media and its reference (when corresponds) is listed in Supplementary Table 1.

The growth curves, doubling time (dt), and the k parameter were compared for *P. salmonis* LF-89 strain in the three media (Figures 2A,B). The dt for *P. salmonis* growing in CMMAB did not differ of the value reached by growing in nutrient-rich media, but it was significantly lower than the dt measured in CMM1, although the k parameter slightly decreased by 17.9%. When considering growth curve dynamics, especially dt and the time to reach the maximum growth, growth curves in CMMAB were more similar to Austral-SRS than to CMM1. Since the nutrient requirement prediction and culture media optimization were developed for LF-89 strain, we aim to evaluate the growth in CMMAB of different *P. salmonis* strains: EM-90 strain, and two strains isolated from more recent SRS outbreaks, CGR02 and Ps12201A. As observed in Figures 2C,D, the four strains showed optimal growth rate in CMMAB media. Moreover, bacterial infectivity was not affected by the medium, as observed by the similar decrease in CHSE-214 cell viability caused by the four bacterial strains grown in Austral-SRS or in CMMAB media (Figure 2E).

### A Genomic Search for Antibiotic Resistance Genes in *Piscirickettsia salmonis*

As antimicrobial susceptibility testing has not been performed using CMMAB medium (or, to our knowledge, any other defined medium) we aim to test *P. salmonis* antibiotic resistance (AR) in a large-scale exploratory approach, without considering previous AR data. Thus, different computational approaches were used in the present study to detect potential ARGs in the genome of the four *P. salmonis* strains (Table 1) and afterward examine the resistance phenotype of the strains.

**TABLE 1:** Summary of bioinformatics tools used for predicting ARGs in the genome of four *P. salmonis* strains.

AMR identifier	Reference database	Prediction method	Selection criteria	Number of ARGs			
				LF-89	CGR02	EM-90	Ps-12201A
SARGfam (Yin et al., 2018)	SARG database v2.0	HMM-based alignment	e-value < 10^–5^	64	64	62	64
RGI (Alcock et al., 2020	Comprehensive Antibiotic Resistance Database CARD 2020	Nucleotide-based homology and SNP models	Loose hits; sequence identity > 0.35	51	47	47	49
AMRFinder (Feldgarden et al., 2019)	Bacterial Antimicrobial Resistance Reference Gene Database	Nucleotide-based homology and HMM-based alignment	All hits	66	67	69	67

*Predicted ARGs are shown for each tool.*

The SARGfam (Structured Antibiotic Resistance Genes v2.0 database with validated ARGs profile HMMs) search was set with strict parameters (e-value < 10^–5^) as recommended. When using the RGI, the selection of “loose” hits allowed detecting new genes or distant ARG homologs. Thus, in this case, we used a cutoff value of 0.35 sequence identity with no query coverage requirements. The AMRFinder algorithm was able to detect only one to two ARGs per genome when the identity and sequence coverage cutoff were set within the recommended parameters. When no cutoff values were set, the number of detected ARGs was similar to that recovered with the other software. With these settings, each bioinformatic tool predicted approximately the same number of ARGs (between 47 and 69 ARGs for *P. salmonis* genome); however, few of them were detected by all three methods employed. As seen in Supplementary Figure 1, only three to seven common genes were predicted as ARGs by the three tools in the four *P. salmonis* strains. However, the SARGfam and AMRFinder tools, which use HMMs search method, predicted a higher number of common ARGs (20 to 22 genes, Supplementary Figure 1).

**FIGURE 3:**
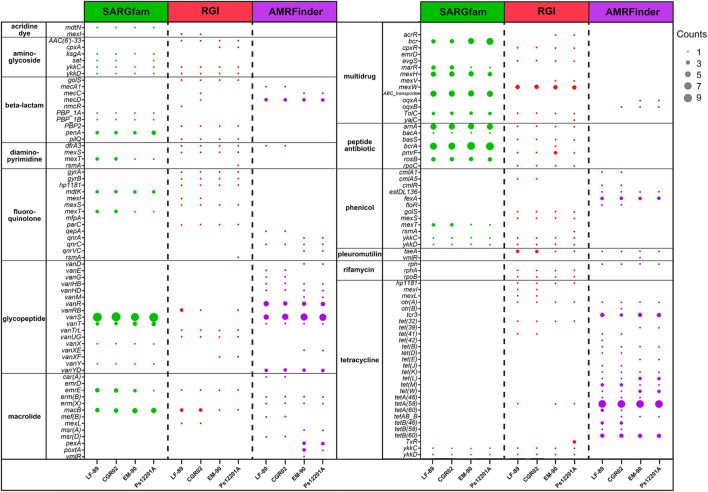
Drug class of predicted ARGs. The number of identified genes for each drug class is shown for *P. salmonis* strains LF-89, CGR02, EM-90, and Ps12201A. The gene counts refer to the number of locus tag predicted with the same ARG name. Note that some ARGs were classified in more than one drug class.

Putative *P. salmonis* ARGs were grouped by resistance mechanism (Supplementary Table 2) and by drug class (Figure 3), following CARD (Comprehensive Antibiotic Resistance Database) classification. Irrespectively of the strain and ARG prediction tool, most of the genes were categorized in antibiotic efflux and antibiotic target alteration resistance mechanisms. Antibiotic target protection also grouped an important number of genes, but this category was tool-dependent, as more ARGs were identified with AMRFinder, followed by RGI, and no genes in this category were found using SARGfam. This search tool dependency also occurred with the antibiotic target replacement category. It is noteworthy that a small number of antibiotic inactivation genes were predicted, suggesting that enzymatic alteration of antibiotics might not be a significant resistance mechanism for *P. salmonis*.

Drug class categories of genes present in more than one strain and predicted with more than one tool are shown in Figure 3. As mentioned, depending on the bacterial strain, between two and seven genes were predicted as ARGs using the three tools (Supplementary Figure 1); however, when arranged in drug class categories as in Figure 3, no gene was observed to be predicted by the three tools. This difference was due to discrepancies in ARG name assignment. For example, the locus tag PSLF89_10 in *P. salmonis* LF-89 was identified as an ARG by SARGfam, RGI, and AMRFinder, but predicted as *penA*, *PBP2*, and *mecD*, respectively. Therefore, one locus tag could be identified as a different ARG by different tools, and several locus tags could have the same ARG name, as occurred with *bcrA*, *vanS*, *macB*, or *tetA(58)* (represented as a bigger dot in Figure 3). Overall, the same drug class ARGs were observed between the strains. The most representative drug class categories across *P. salmonis* strains were selected to conduct phenotypic assays, as we speculated that the higher number of ARGs predicted for a specific antibiotic could make a resistance phenotype more probably observed.

### Antibiotic Susceptibility Phenotypes of *Piscirickettsia salmonis*

To validate the resistance phenotypes that were computationally predicted in the genome of the *P. salmonis* strains, a panel of antibiotics of the most represented drug classes were tested for antimicrobial susceptibility performing MIC tests, according to CLSI standards. For MIC testing, we used the optimized defined media broth CMMAB and the Austral-SRS medium which is the recommended medium by CLSI guidelines (Miller et al., 2014). Results are shown in Table 2. MIC data are displayed as range values to depict more accurately what was observed in the assay plates due to the variability of MIC values. For some antibiotics, such as tetracyclines, erythromycin, florfenicol, and ampicillin, MIC values were not always constant and could vary between one-fold and four-fold dilutions in a medium-independent fashion. The initial assumption was that CMMAB medium, by containing adjusted cations, will provide more stable MIC values as they will not depend on membrane permeability (Nikaido, 2003; Hawke et al., 2005), but the results suggested that the variability was an intrinsic property of the bacteria. All tested *P. salmonis* strains were found to be resistant to polymyxin B, vancomycin, ceftazidime, and trimethoprim. Interestingly, the susceptibility to the antibiotics erythromycin, ampicillin, penicillin G, streptomycin, and spectinomycin was medium-dependent, showing higher MIC values in the CMMAB media. Of note, the four *P. salmonis* strains were found to be susceptible to the antibiotics of therapeutic significance, with MIC values for oxytetracycline ranging from 0.08 to 0.32 μg/ml in Austral-SRS and 0.02 to 0.32 μg/ml in CMMAB, and MIC values for florfenicol between 0.04 and 0.64 μg/ml in Austral-SRS and 0.08–1.28 μg/ml in CMMAB.

**TABLE 2:** MIC values (μg/ml) observed for *P. salmonis* strains in nutrient broth (Austral-SRS) and the defined medium (CMMAB).

		LF-89				CGR02				EM-90				Ps12201A			
		
		MIC range		Phenotype		MIC range		Phenotype		MIC range		Phenotype		MIC range		Phenotype	
		
Drug family	Antibiotic name	Austral-SRS	CMM-AB	Austral-SRS	CMM-AB	Austral-SRS	CMM-AB	Austral-SRS	CMM-AB	Austral-SRS	CMM-AB	Austral-SRS	CMM-AB	Austral-SRS	CMM-AB	Austral-SRS	CMM-AB
Tetracyclines	Tetracycline	0.16–0.32	0.02–0.08	S	S	ND	ND	ND	ND	ND	ND	ND	ND	ND	ND	ND	ND
	Oxytetracycline^§^	0.16–0.32	0.02–0.08	S	S	0.08–0.32	0.02–0.08	S	S	0.08–0.16	0.02–0.16	S	S	0.08–0.16	0.02–0.08	S	S
Fluoroquinolone, quinolone antibiotic	Flumequine^§^	0.32–0.64	0.08–0.16	S	S	0.08–0.64	0.02–0.16	S	S	0.04–0.08	0.02–0.04	S	S	0.04–0.16	0.02	S	S
	Oxolinic acid	0.32	0.04–0.08	S	S	0.32–0.64	0.08–0.16	S	S	0.04–0.16	0.02–0.16	S	S	0.04–0.08	0.02	S	S
Macrolide antibiotic	Erythromycin	1.6–6.4	12.5–25	S	R	1.6–12.5	3.12–12.5	S	I	0.4–1.6	3.12–12.5	S	I	0.8–1.6	12.5–25	S	R
Phenicol antibiotic	Chloramphenicol	0.16–0.64	0.16–0.32	S	S	ND	ND	ND	ND	ND	ND	ND	ND	ND	ND	ND	ND
	Florfenicol^§^	0.08–0.32	0.32–0.64	S	S	0.04–0.64	0.08–1.28	S	S	0.08–0.32	0.312–1.25	S	S	0.08–0.32	0.32–1.28	S	S
Peptide antibiotic	Polymyxin B	6.25–12.5	12.5–25	I	R	12.5	6.25–12.5	R	I	12.5	25–125	R	R	12.5–25	12.5–50	R	R
Glycopeptide antibiotic	Vancomycin*	>1,000	>1,000	R	R	>1,000	>1,000	R	R	>1,000	>1,000	R	R	>1,000	>1,000	R	R
Beta-lactam, cephalosporin	Ampicillin	0.1–0.8**	200–400	S	R	0.1–0.4**	125–250	S	R	0.08–0.4**	31.2	S	R	0.08–0.4**	31.2–250	S	R
	Penicillin G	0.4–0.8**	25–200	S	R	ND	ND	ND	ND	ND	ND	ND	ND	ND	ND	ND	ND
	Ceftazidime	>1,000**	>1,000**	R	R	>1,000**	>1,000**	R	R	>1,000**	>1,000**	R	R	>1,000**	>1,000**	R	R
Aminoglycoside antibiotic	Streptomycin	6.25–12.5	50–100	I	R	3.12–6.25	100–400	S	R	6.25–12.5	400	I	R	6.25	100–400	S	R
	Spectinomycin	6.25–25	12.5–50	I	R	ND	ND	ND	ND	ND	ND	ND	ND	ND	ND	ND	ND
Diaminopyrimidine antibiotic	Trimethoprim	125	250	R	R	62.5–125	500	R	R	62.5–125	500	R	R	125	250–500	R	R
Rifamycin antibiotic	Rifampicin	1.6	0.8–1.56	S	S	1.6	0.8–1.6	S	S	0.8–1.6	0.8–1.6	S	S	0.8–1.6	0.8–1.6	S	S

*Bacteria were considered resistant (R) to an antibiotic if MIC > 10 μg/ml; if not, they were labeled as susceptible (S). If MIC range was around the breakpoint of the resistant phenotype, then an intermediate range (I) of susceptibility was defined.*

*^§^ Antibiotics used in aquaculture.*

*Antibiotic for Gram-positive bacteria.

**Suboptimal growth compared to the control.

Vancomycin, an antibiotic for Gram-positive bacteria, was included in the MIC tests even though *P. salmonis* is Gram-negative, due to the high number of ARGs targeting vancomycin that were predicted with different tools. A total of 25 vancomycin resistance genes, corresponding to 18.2% of all ARGs, were identified in *P. salmonis* LF-89. As shown in Table 2, the level of resistance to vancomycin in *P. salmonis* was so high that it could be attributable to its Gram-negative nature, rather than the presence of these putative vancomycin-resistant genes. This supports the importance of manually curing the bioinformatics results and contrasting the bioinformatic predictions obtained by current available tools with phenotypic data.

**FIGURE 4:**
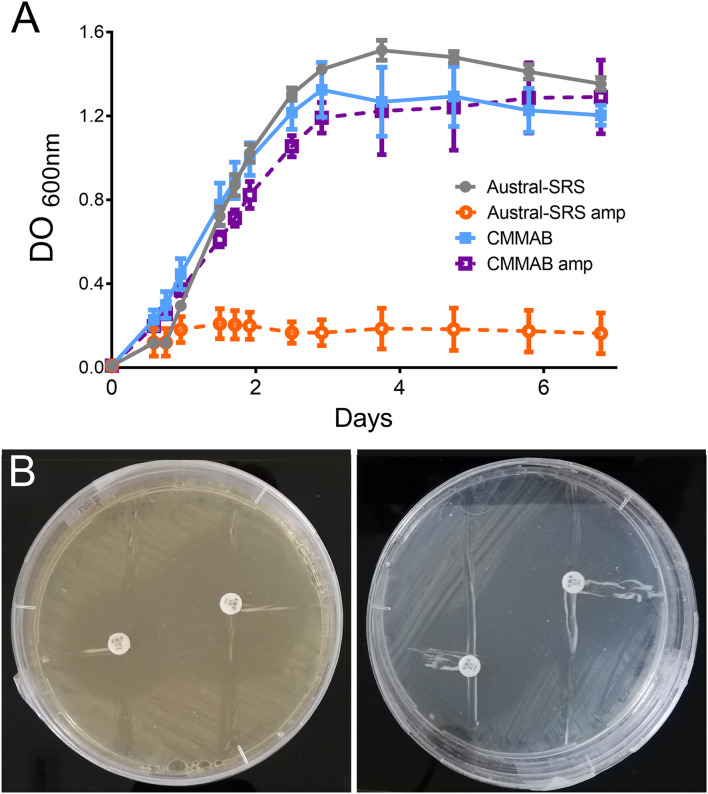
*P. salmonis* LF-89 response to ampicillin. **(A)** *P. salmonis* growth curves in nutrient (Austral-SRS) and optimized defined (CMMAB) culture media with and without ampicillin (amp). **(B)** *P. salmonis* clover-leaf test for ampicillin. *E. coli* DH5α was used as the indicator strain. *E. coli* and *P. salmonis* were grown in Austral-SRS (left) and CMMAB (right) agar plates with disks containing 100 mg and 500 mg of amp (small and large inhibition zone, respectively).

According to Table 2, *P. salmonis* showed decreased susceptibility to ampicillin (amp) in the CMMAB medium compared to other tested antibiotics (resistant to >200 μg/ml of amp in MIC) and presented a higher difference in MIC values between both growth media (500- to 2000-fold). Thus, we sought to investigate the possible mechanism underlying the medium-dependent amp resistance in *P. salmonis*. As MIC tests are end-point experiments, we aimed to further evaluate the bacterial behavior by recording *P. salmonis* strain LF-89 growth curves (Figure 4A). Amp addition to the culture media impaired *P. salmonis* growth only in the nutrient media (Austral-SRS amp), as expected, whereas normal growth in CMMAB amp could be recorded.

Given that β-lactamase activity has been previously suggested for other *P. salmonis* strains (Mikalsen et al., 2007), and one ARG with antibiotic inactivation resistance mechanism was predicted in the LF-89 strain, we evaluated this possibility by performing clover-leaf tests. Agar Austral-SRS and CMMAB media allowed *P. salmonis* LF-89 growth, but no evidence of β-lactamase production was detected (Figure 4B).

**FIGURE 5:**
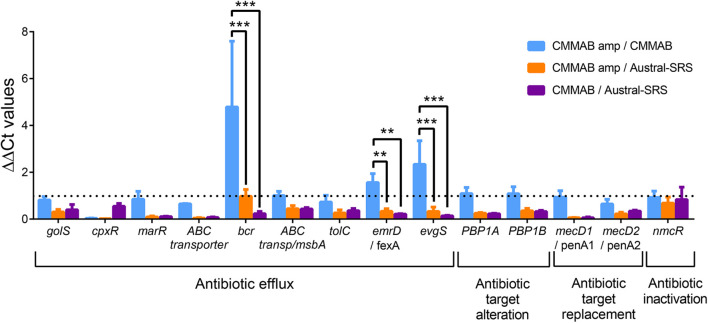
Expression of putative amp resistance genes in *P. salmonis* grown in the different media. Relative transcript abundance of selected ARGs predicted to confer amp resistance in *P. salmonis* LF-89. Bar-graph showing transcript levels quantified by qPCR and expressed as ΔΔCt values normalized by the housekeeping genes *glyA* and *pykA*. The dotted line indicates the normalized control values (ΔΔCt = 1). A two-way ANOVA with Tukey’s test for multiple comparisons was performed. Significantly different values are marked (***p < 0.001 and **p < 0.01).

**FIGURE 6:**
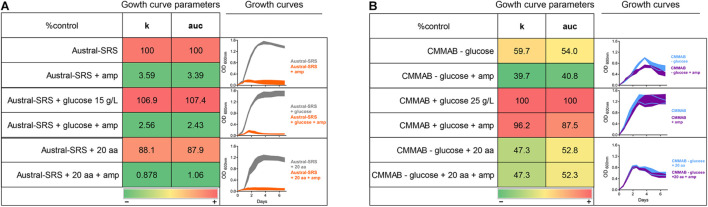
Effect of glucose and amino acid supplements in amp resistance. *P. salmonis* LF-89 growth curves and curve parameters (carrying capacity k and area under the curve auc) in Austral-SRS nutrient medium **(A)** and the optimized defined medium CMMAB **(B)** with or without glucose and amino acid supplements and their effect in bacterial resistance to amp. Note that “20 aa” refers to the mixture of amino acids used in the CMM1 composition, and in the case of CMMAB, they were added in addition to the amino acids present in the medium.

To further investigate the resistance mechanism to amp, expression levels of selected ARGs were evaluated in *P. salmonis* strain LF-89 during exponential growth phase in both culture media, with or without the mentioned antibiotic (Figure 5). Since *P. salmonis* was not able to grow in the Austral-SRS medium with amp, the antibiotic was used only in the CMMAB medium and cultures with no antibiotic in both media were used as controls. The only gene predicted to inactivate amp, *nmcR*, was not overexpressed in the presence of amp, which correlated with the negative clover-leaf test. In addition, all antibiotic target alteration and antibiotic target replacement genes were not significantly overexpressed in the CMMAB amp or in the CMMAB media. Three antibiotic efflux genes were upregulated in the presence of the antibiotic, but only in the comparison between CMMAB amp and CMMAB media, whereas the expression levels were similar between bacteria grown in CMMAB amp and in Austral-SRS. The observed fold change in ARGs expression levels does not explain the radically different MIC values observed between both culture media. Thus, we speculated that media composition could be relevant for the resistant phenotype observed in *P. salmonis*, and the bacterial resistance to amp was not the result of overexpression of the predicted ARGs. To test that possibility, nutrient composition was changed in the culture media and growth parameters were compared. As shown in Figure 6A, neither glucose nor aa supplementation could recover *P. salmonis* growth in Austral-SRS medium, suggesting that addition of these nutrients is not sufficient to increase bacterial resistance. In contrast, the growth parameters of *P. salmonis* in CMMAB medium without glucose or with an excess of aa did not differ after exposing to ampicillin, indicating that *P. salmonis* remained resistant to amp in this defined medium (Figure 6B). This result also suggests that nutrient scarcity may play a role in the bacterial antibiotic resistance.

### Evaluation of *Piscirickettsia salmonis* Transcriptome Changes by RNA-Sequencing

**FIGURE 7:**
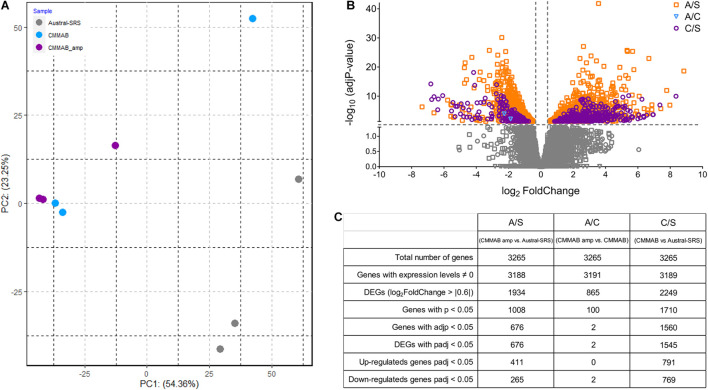
RNA sequence data for *P. salmonis* LF-89. **(A)** Principal component analysis (PCA) plot of gene expression profiles of bacteria grown in Austral-SRS, CMMAB, or CMMAB with amp. **(B)** Gene transcripts identified in the following comparisons: bacteria grown in CMMAB with amp vs. Austral-SRS (A/S, squares); bacteria grown in CMMAB with amp vs. CMMAB (A/C, inverted triangles); and bacteria grown in CMMAB vs. Austral-SRS (C/S, circles). Data between and below the dotted lines (marked in gray) were not statistically significant (adjusted *p*-value > 0.05, log_2_ fold change < | 0.6|). **(C)** Table showing a summary of the number of genes identified after RNA sequencing and data processing.

A transcriptomic analysis was performed to elucidate the molecular mechanisms of *P. salmonis* resistance to amp. *P. salmonis* transcriptomes were named as follows: sample S (*P. salmonis* grown in Austral-SRS), sample C (*P. salmonis* grown in CMMAB), and sample A (*P. salmonis* grown in CMMAB with amp), and three sets of biological replicates per sample were sequenced. We used an unsupervised classification method, principal component analysis (PCA), to characterize the differences between the gene expression profiles of the nine samples. Figure 7A shows that three of the biological replicates of sample A and two replicates of sample C grouped together and separated from the replicates of sample S, especially along the PC1 axis, which explains 54.36% of the variation. This result indicates that CMMAB and Austral-SRS media induced a gene expression response that differed over a wide range of genes, while the amp treatment did not produce additional changes in the gene expression levels. To assess these differences, we compared the transcriptome of *P. salmonis* growing in CMMAB (with or without amp) and in the Austral-SRS medium. A total of 1,545 genes were differentially expressed in the CMMAB medium with amp compared to the Austral-SRS medium (A/S), and 676 genes were differentially expressed in CMMAB without ampicillin compared to Austral-SRS (C/S), as shown in Figures 7B,C. In the CMMAB medium, most differentially expressed genes were upregulated in comparison to the Austral-SRS (60.8% of DEGs in C/S), whereas 51.2% of DEGs were upregulated in the A/S comparison (Figure 7B). However, only two genes were differentially expressed between *P. salmonis* growing in the CMMAB media with and without ampicillin (A/C), and both genes (a hypothetical protein with CBS domain and a universal stress family protein) were downregulated in response to amp (Supplementary Table 3). Thus, the *P. salmonis* gene profile revealed that the presence of amp in the CMMAB medium had a marginal impact on gene expression; however, major expression changes took place when the bacteria were grown in CMMAB as compared to the nutrient-rich medium Austral-SRS (Figure 7B). As an independent measure of differential gene expression, we examined the relative expression of 14 genes derived from qPCR assays presented in Figure 5 and compared them with the RNA-seq transcript levels (Supplementary Figure 2). Overall, a positive correlation between 0.58 and 0.87 (Pearson correlation) was determined between qPCR and RNA-seq for the combined data set (*p* < 0.01).

**FIGURE 8:**
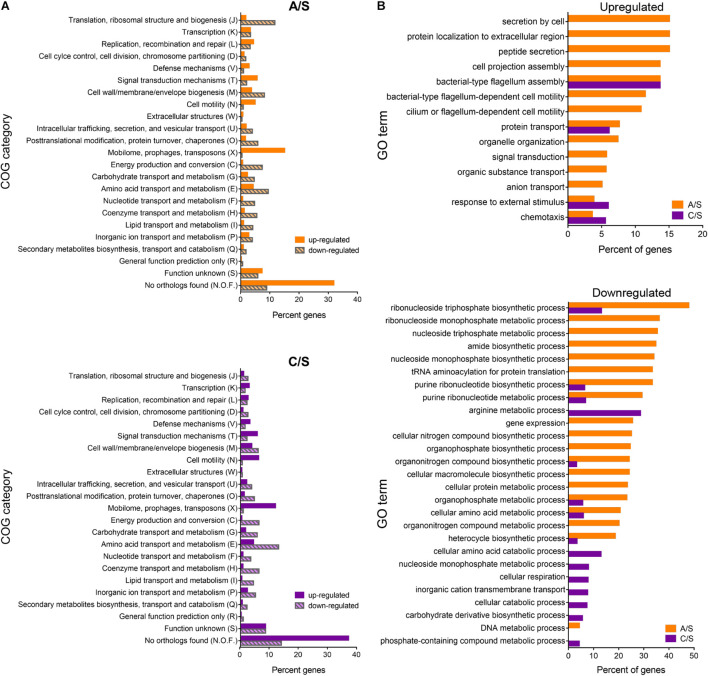
Differentially expressed genes classified into biological and functional gene categories. **(A)** Functional categories of *P. salmonis* genes. Percent of differentially expressed genes in each COG (Cluster of Orthologous Groups) identified by total RNA sequencing for the exponentially growing bacteria in the comparisons CMMAB with ampicillin vs. Austral-SRS (A/S, orange), and CMMAB without ampicillin vs. Austral-SRS (C/S, purple). **(B)** Enrichment analysis of differentially expressed genes. Percent of genes categorized in significantly enriched GO terms are shown for up- and downregulated genes (top and bottom panels, respectively).

The differentially expressed genes were classified into biological and functional gene categories according to the Cluster of Orthologous Groups (COG) and Gene Ontology (GO) databases. COG categories assigned to A/S and C/S up- and downregulated genes are shown in Figure 8A. In general, most upregulated genes in A/S and C/S were classified in the categories mobilome, prophages, transposons, signal transduction mechanisms, and cell motility (X, T, and N, respectively). Downregulated genes, on the other hand, belonged to translation, and ribosomal structure and biogenesis categories for the A/S comparison, and to amino acid transport and metabolism, energy production and conversion, and cell wall/membrane/envelope biogenesis (E, C, and M, respectively) for both comparisons. Interestingly, most of the upregulated genes in A/S and C/S comparisons were unique to *P. salmonis* as they encoded hypothetical proteins with unknown orthologs (N.O.F. in the bar graphs, Figure 8B), suggesting that *P. salmonis* growth in the CMMAB medium triggers unknown adaptation mechanisms that are unique for this bacterium.

Enrichment analysis revealed significantly overrepresented biological processes in the dataset of differentially expressed genes (Figure 8B and Supplementary Table 4). Overrepresented GO terms in both comparisons were related to flagellum assembly and organization, chemotaxis, and response to external stimulus. In the A/S comparison, protein secretion, transport of peptides, amides, anions, nitrogen compounds, and organic substances were also overrepresented. It is worth noticing that genes belonging to these categories were also upregulated in the C/S comparison, although these GO terms did not achieve statistical significance after enrichment analysis. Some of the upregulated genes annotated to the term chemotaxis were related to flagellar- and/or cilium-dependent cell motility, whereas others were nutrient-sensing or nutrient transport mechanisms, such as the *phoQ* sensor histidine kinase (PSLF89_3272), sodium:proton exchanger family proteins (PSLF89_2485 and PSLF89_2203), and ABC-type amino acid transport signal transduction systems (PSLF89_1726 and PSLF89_1196). Although genes encoding the sensor proteins were upregulated, several sodium exchanger-type transporters and permeases were downregulated, such as a calcium:proton antiporter (PSLF89_2152), an acetylneuraminate ABC transporter (PSLF89_803), the proline:sodium symporter *putP* (PSLF89_1458), sodium:dicarboxylate symporter (DAACS) family protein (PSLF89_2288 and PSLF89_725), an aromatic amino acid transport family protein (PSLF89_3306), and various amino acid permeases (PSLF89_3168, PSLF89_2573, PSLF89_180 and PSLF89_817). This correlates with an underrepresentation of GO terms related to transmembrane transport (C/S comparison), and amino acid metabolic processes (C/S and A/S comparisons), which was indicative of a general metabolic downshift represented by a downregulation of the GO term generation of precursor metabolism and energy (C/S). In addition, numerous biosynthetic and metabolic processes were significantly downregulated in CMMAB and CMMAB amp media compared to Austral-SRS, especially those related to nucleotide, ribonucleotide, nucleoside, and ribonucleoside metabolic and biosynthetic processes (Figure 8B). Interestingly, A/S comparison differed from the C/S comparison in a downregulation of GO terms related to DNA metabolic process, gene expression, translation, and nucleobase metabolism and biosynthesis.

**FIGURE 9:**
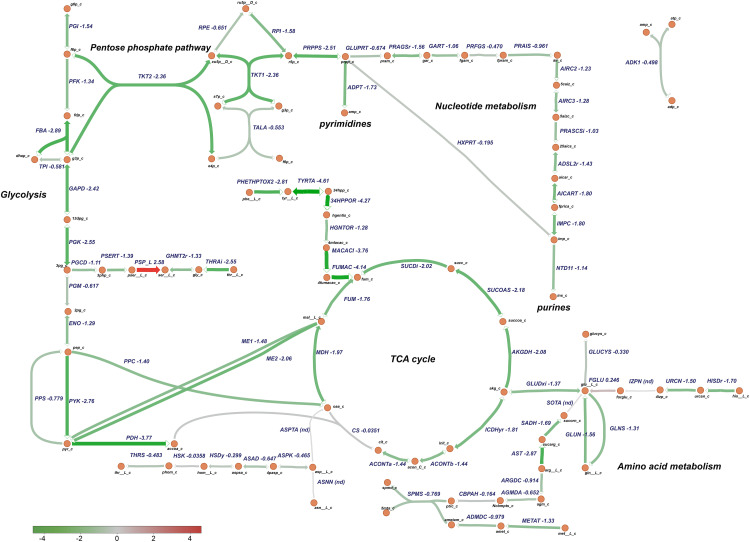
Downregulation of central metabolic pathways in *P. salmonis* growing in CMMAB with amp. Genes differentially expressed between *P. salmonis* grown in the CMMAB amp medium and the Austral-SRS medium were mapped to glycolysis, the TCA cycle, pentose phosphate pathway, nucleotide, and amino acid metabolism using the genome-scale model iPS584 for this pathogen. Expression levels are shown as log_2_ fold-change values; downregulated genes are in green and upregulated genes are in red.

**FIGURE 10:**
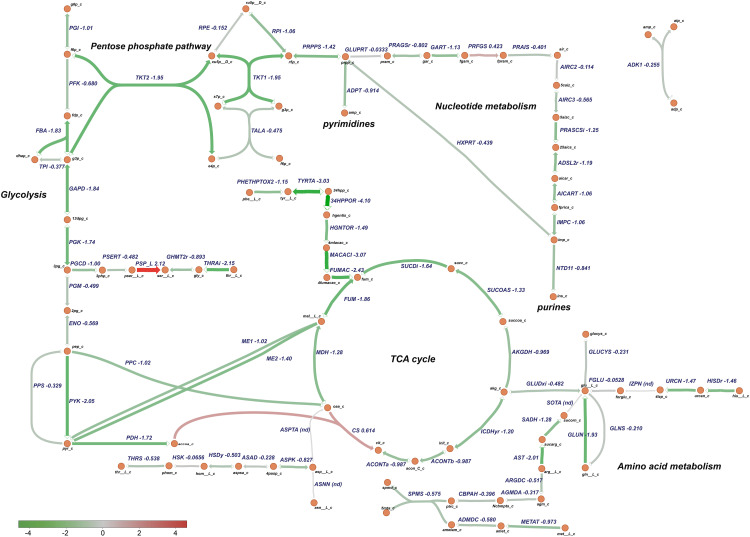
Downregulation of central metabolic pathways in *P. salmonis* in CMMAB medium. Genes differentially expressed between *P. salmonis* grown in the CMMAB medium without amp and the Austral-SRS medium were mapped to the TCA cycle, pentose phosphate pathway, nucleotide, and amino acid metabolism using the genome-scale model iPS584. Expression levels are shown as log_2_ fold-change values; downregulated genes are in green and upregulated genes are in red.

Based on these analysis, transcriptomic data were mapped to specific *P. salmonis* metabolic pathways using a genome-scale model previously reconstructed for this organism (Cortés et al., 2017). Particularly, metabolism of nucleotides, amino acids, glycolysis, and the TCA cycle were selected for analysis based on the categories associated to the differentially expressed genes (Figure 8). Metabolic pathway analysis revealed an overall downregulation of genes belonging to those processes, in the bacteria growing in the CMMAB medium with or without amp when compared to Austral-SRS medium (Figures 9, 10 and Supplementary Table 4), with fold changes being consistently smaller when the bacteria was exposed to the antibiotic. A selection of differentially expressed genes with significant fold-change values as shown in Figures 9, 10 were subjected to validation by comparing the normalized gene expressed levels (TMM+CPM) between the three samples (Supplementary Figure 3). Genes from relevant pathways in Figures 9, 10, such as amino acid metabolism, nucleotide metabolism, TCA cycle, pentose phosphate pathways and glycolysis, were significantly downregulated in A and C samples (bacteria grown in CMMAB + amp and CMMAB, respectively) when compared to S samples (bacteria grown in Austral-SRS), with the exception of genes coding for PSP_L (amino acid metabolism), CS (TCA cycle), and PRFGS (nucleotide metabolism) enzymes, which were consistently found overexpressed in A and C when compared to S (red arrows).

*Piscirickettsia salmonis* exhibited lower expression of genes associated with glycolysis, even in reactions such as phosphofructokinase (PFK, PSLF89_1090) and fructose-bisphosphate aldolase (FBA, PSLF89_1028), which are associated with fructose 6 phosphate and fructose-1,6-bisphosphate, both of which are entry points of the pentose phosphate pathway (PPP). PPP provides precursors for the synthesis of nucleotides, which were also downregulated in *P. salmonis* growing on CMMAB with or without ampicillin (A/S and C/S). Downregulation of genes encoding components of the pyruvate node, a key point of regulation between glycolysis and the TCA cycle was also observed. Genes encoding pyruvate kinase (PYK, PSLF89_1027), pyruvate dehydrogenase (PDH, PSLF89_3190, PSLF89_3189, PSLF89_3188, PSLF89_2098, PSLF89_3189, and PSLF89_3190), and malic enzymes (ME1, PSLF89_3304, ME2, and PSLF89_234) showed lower expression levels in the CMMAB medium with or without amp compared to Austral-SRS medium. For the TCA cycle, a decrease in gene expression was observed for all the reactions in the CMMAB medium, with the exception of citrate synthase (CS, PSLF89_893). Genes encoding enzymes of the synthesis of glutamine, an amino acid that can also fuel the TCA cycle, were also downregulated [glutaminase (GLUN, PSLF89_1767, and PSLF89_307), glutamine synthase (GLNS, PSLF89_179), and glutamate dehydrogenase (GLUDxi, PSLF89_2806)]; in this case, greater downregulation was observed in CMMAB medium supplemented with amp.

## Discussion

### *Piscirickettsia salmonis* Nutrient Requirements

Two previous works have described *P. salmonis* nutritional requirements based on genomic reconstructions of the bacterial metabolism (Cortés et al., 2017; Fuentealba et al., 2017). Recently, Fuentealba et al. (2020) used these metabolic predictions to improve a defined medium intended to be used in vaccine development. In this wok, we created a new defined culture media that contains 14 essential amino acids for *P. salmonis* growth. As previous works stated, we observed the importance of glucose and amino acids as carbon sources in the media, although comparison of growth curve parameters revealed the importance of NaCl. The defined media described in Cortés et al. (2017) did not allow for *P. salmonis* growth without glucose as a carbon source; in contrast, Fuentealba et al. (2017) reported that a defined media with a mixture of 12 aa and no glucose or other carbon sources allowed bacterial growth. Both defined media were composed of amino acids (20 aa in Cortés’s defined media, and 12 aa in Fuentealba’s media), vitamins, salts, and an iron source, but the main difference between them was the presence of NaCl and the absence of glucose in Fuentealba’s media. Based on our results, the CMM1 media (similar to Cortés’s defined media composition) without glucose allowed a 33.8% of the growth obtained in Austral-SRS, but by adding NaCl to the medium, the growth of *P. salmonis* increased to reach levels similar to those measured in the nutrient-rich medium (Figure 1A). Fuentealba’s work described that 16% of *P. salmonis* transporters were specific for aa and peptides (Fuentealba et al., 2017), thus accounting for a greater capacity to assimilate amino acids from the surrounding environment. This observation, together with the presence of sodium/amino acid symporters in *P. salmonis* genome and our results on the effect of NaCl on *P. salmonis* growth, suggests that the capability of incorporating aa to the cell is related to the presence of NaCl in the medium.

The auxotrophy tests carried out in this work yielded similar results to those obtained by Fuentealba et al. (2020). Thus, arginine, isoleucine, leucine, lysine, methionine, proline, phenylalanine, threonine, and valine were needed for *P. salmonis* growth. Since *P. salmonis* is auxotrophic for the amino acids arginine, cysteine, histidine, isoleucine, leucine, methionine, and valine, and they are therefore strict nutritional requirements, the rest of the amino acids seem to be necessary to improve bacterial growth in the CMMAB medium. Improvement of growth allowed narrowing down the cultivation time, while still attaining growth levels similar to those observed in a nutrient-rich medium. Coincidences exists between the amino acid auxotrophies of *P. salmonis*, *L. pneumophila* (Tesh and Miller, 1981), and *C. burnetii* (Sandoz et al., 2016), two other intracellular bacteria often regarded as closely related to *P. salmonis*. Both *L. pneumophila* and *C. burnetii* exploit the amino acid pools of the host while replicating intracellularly and greatly rely on amino acids as a main source of carbon and energy (Price et al., 2014; Sandoz et al., 2016). Besides a previous work reporting the expression of a number of genes associated with the amino acid metabolism in *P. salmonis* and *S. salar* during infection (Valenzuela-Miranda and Gallardo-Escárate, 2018), the study of the metabolic connection between *P. salmonis* and its host remains poorly explored. Therefore, through the analysis of the nutritional requirements that promote the intracellular lifestyle of *P. salmonis*, it is expected that the CMMAB medium presented here could contribute to understand how *P. salmonis* exploits the host metabolism to replicate and survive in the intracellular milieu.

On the other hand, our results showed that *P. salmonis* growth was partially inhibited by supplementation with alanine, asparagine, glutamine, tryptophan, and tyrosine, an observation that was also in agreement with the work of Fuentealba et al. (2020). Inhibition of bacterial growth by amino acids seems to be caused by feedback inhibition of an early enzyme in the pathway of the amino acid synthesis; this inhibition in turn represses the production of other amino acids that use the common enzyme for their syntheses. The inhibitory effect of amino acids on bacterial growth has been known for many years, and growth inhibitions have been reported for *Streptococcus bovis* (Washburn and Niven, 1948), *E. coli* (Rowley, 1953), and *Chlamydia trachomatis* (Al-Younes et al., 2006), among other bacteria.

In our media composition, we included glucose as a carbon source additional to amino acids. In this condition, we observed that aspartic acid, glycine, and serine supplementation improved bacterial growth parameters, whereas glutamic acid decreased bacterial growth as opposed to what was described for the vaccine formulation in the absence of glucose (Fuentealba et al., 2020). In this context, our defined medium CMMAB improved bacterial growth to levels observed in the nutrient-rich medium (Austral-SRS), buffered the pH increasing over time, and sustained bacterial infectivity in the CHSE-214 cell line model; accordingly, cytopathic effects were indistinguishable from the ones observed when the bacterium was grown in Austral-SRS medium.

### *Piscirickettsia salmonis* Antibiotic Resistance Prediction and Phenotypes

Previous studies have reported *P. salmonis* resistance to penicillin by disk diffusion assays (Mikalsen et al., 2007), and to penicillin and spectinomycin by MIC assays in cell cultures (Cvitanich et al., 1991). On the other hand, moderate degrees of susceptibility to florfenicol, oxytetracycline, flumequine, and oxalinic acid have been reported in MIC assays using the Austral-SRS media (Yánez et al., 2013; Henríquez et al., 2016; Contreras-Lynch et al., 2017). A controversy was generated regarding the MIC assessment and its relation to the epidemiological cutoff values (ECOFF) for florfenicol and oxytetracycline, both antibiotics profusely used in Chilean aquaculture (Mancilla, 2018), as two works reported similar MIC values but different interpretation of bacterial population distribution based on MIC results. While Contreras-Lynch et al. (2017) concluded that the *P. salmonis* population did not considerably decrease its susceptibility to florfenicol and oxytetracycline, Henríquez et al. (2016) reported a bacterial population of concern with decreased susceptibility to this antibiotics, although both works reported similar MIC values for the tested *P. salmonis* strains (Henríquez et al., 2016; Contreras-Lynch et al., 2017). Here, we obtained the MIC values for only four strains (in contrast to the 58 and 292 isolates reported previously) (Henríquez et al., 2016; Contreras-Lynch et al., 2017), and based on our cutoff value (10 μg/ml), they were classified as susceptible to the antibiotics used in aquaculture (Table 2). Moreover, considering the previously reported MIC values, all *P. salmonis* isolates tested in the Henríquez et al. (2016) and the Contreras-Lynch et al. (2017) reports fall within the susceptible categories defined in this work. On the other hand, when growing in the CMMAB medium, *P. salmonis* prove to be resistant to 9 of the 16 antibiotics tested, and in contrast, the bacterium was resistant to only three antibiotics in the Austral-SRS medium, thus revealing the importance of the culture media composition for antibiotic resistance testing in *P. salmonis*. To our knowledge, differential resistance phenotypes depending on the growth media have not been previously reported in *P. salmonis*.

Gene variations and gene mutations conferring antibiotic resistance have been the primary way to explain bacterial resistance to antibiotics (Espedido and Gosbel, 2012). Here, more than 130 individual genes were predicted as ARGs in the four strains, and 14 of them could be related to ampicillin resistance. Considering that ARG databases have been constructed based on genomes and gene information from human-related pathogens (Feldgarden et al., 2019), discrepancies between predicted ARGs and resistance phenotypes were expected as the search parameters used in this work privileged encountering distant ARG homologs in a fish pathogen. Therefore, this ARG prediction with loose parameters needed an important experimental counterpart to test antibiotic resistance potential. Predicted ARGs belonging to the categories of antibiotic target alteration and antibiotic target replacement resistance mechanism were expressed in all tested media, but with similar expression levels. This was a possible result since these types of ARGs may not be inducible by amp and thus exert their function just by expressing, and not by overexpressing (Munita and Arias, 2016). In contrast, antibiotic inactivation and antibiotic efflux genes were expected to be overexpressed in the CMMAB amp medium; however, gene expression changes were detected only between the bacteria grown in CMMAB medium and not between CMMAB amp and Austral-SRS. Thus, changes in ARG expression levels did not explain the 500- to 2000-fold increase in MIC observed between Austral-SRS and CMMAB media. In addition, the lack of a positive result in the clover-leaf test for ampicillin supports the predicted ARGs gene expression results, and therefore, no phenotypic evidence of antibiotic inactivation was observed in our experimental conditions.

In our conditions, addition of glucose or aa in the nutrient-rich media did not decrease *P. salmonis* susceptibility to amp, suggesting that the presence of these nutrients was not the sole requirement that allowed bacterial growth. On the contrary, nutrient scarcity in the defined CMMAB media seemed to provide the adequate conditions for bacterial growth in the presence of the antibiotic. Media composition for antibiotic susceptibility testing should be considered when studying pathogenic bacteria, as media mimicking host environments (Ersoy et al., 2017) have proven to be more reliable to predict antibiotic efficacy in the host.

### Metabolic Resistance as a Mechanism for *Piscirickettsia salmonis* Ampicillin Resistance

Since culture media composition can have a significant impact on bacteria metabolic state and global physiological changes can occur as a consequence of antibiotic exposure (Yang et al., 2017, 2019; Shin et al., 2021), we reasoned that a more global bacterial response, rather than the induction of a few genes, needed to be analyzed in order to elucidate the amp resistance mechanism in *P. salmonis*. Transcriptomic analysis showed that *P. salmonis* growing in the CMMAB medium (and CMM amp medium) senses specific external stimulus, such as particular nutrients, that triggers signal transduction mechanisms. Some of them were chemotaxis-related genes and flagellar- and/or cilium-dependent cell motility. As stated in previous works, *P. salmonis* does not possess a functional flagellum that confers motility in any examined condition (Cvitanich et al., 1991; Mauel and Miller, 2002), although flagellar proteins and genes are expressed during intracellular infection (Ortiz-Severín et al., 2020; Zúñiga et al., 2020), which suggests an alternative role to flagellar structures during infection and intracellular growth (Machuca and Martinez, 2016), and now, during growth in nutrient scarcity conditions in a defined media.

Although CMMAB media contains sufficient nutrients to allow bacterial growth in similar levels to those obtained with the nutrient-rich media (Figures 2A,B), results indicate that *P. salmonis* suffers a metabolic downshift when grown in the CMMAB medium that coincides with nutrient deprivation. As Austral-SRS media contains an excess of nutrients with complex carbon sources, including a soybean casein digest and fetal bovine serum, the sole presence of amino acids, glucose, and salts in CMMAB medium constitutes a decrease in nutrient availability for the bacterium. As described for *E. coli* when growth is nutrient-limited in the presence of an excess of energy, nutrient availability is correlated with growth but not with the metabolism (Erickson et al., 2017; Lopatkin et al., 2019), and the excess of available energy goes to cellular activities other than growth. In this condition, *E. coli* survival to treatments with bactericidal antibiotics depends on cellular metabolism irrespective of the growth rate, as accelerated respiratory activity potentiates antibiotic lethality (Lopatkin et al., 2019). Although a link between cell death caused by bactericidal antibiotics and accelerated respiration was previously stablished (Lobritz et al., 2015), the role of growth rate was unclear. Here, we observed similar growth parameters in Austral-SRS and CMMAB media, but considerably different MIC were observed for several bactericidal antibiotics. Thus, we hypothesize that in *P. salmonis* growing in CMMAB, the increased MIC for amp responds to a change in the metabolic state of the bacterium, although further experiments need to be conducted to support this idea.

Lethality by bactericidal antibiotics, such as amp, has been associated with a build-up of toxic metabolic by-products as a consequence of an overly active central carbon metabolism and increased abundance of central carbon metabolites (Belenky et al., 2015; Stokes et al., 2019). These perturbations led to an oxygen consumption, breakdown of nucleotides, and induction of redox stress (Belenky et al., 2015). In addition, TCA cycle metabolites, aa, and nucleotides activate bacterial metabolism, and in turn, a decreased TCA, aa, and nucleotide metabolism, such as observed in *P. salmonis* grown in CMMAB and CMMAB amp, is correlated with a decreased proton-motive force that can drive antibiotic incorporation by the cell, and overall respiration that increases cell death in the presence of antibiotics (Lobritz et al., 2015; Stokes et al., 2019; Liu et al., 2020).

Recently, Lopatkin et al. (2021) replicated metabolic mutations found in antibiotic resistance *E. coli* strains, obtaining antibiotic-tolerant strains as a result. Two of these metabolic mutations were associated to the TCA cycle [2 oxoglutarate dehydrogenase (AKGD) and isocitrate dehydrogenase (ICDHyr)], and one of them was associated with amino acid metabolism (glutamate synthase). In this work, an overall downregulation of central carbon metabolism is observed in CMMAB media. Particularly, both AKGD (PSLF89_3188, PSLF89_898, and PSLF89_899)- and ICDHyr (PSLF89_2677)-associated transcripts were downregulated compared to the control condition in which antibiotic resistance is not observed. We propose that downregulation of these genes provides a metabolic context, given by nutrient scarcity, that protects *P. salmonis* against the detrimental effects of amp, thus suggesting a metabolic regulation of antibiotic resistance in *P. salmonis*.

## Materials and Methods

### Bacterial Strains

The reference strain LF-89 (ATCC VR-1361) was obtained from the American Type Culture Collection (ATCC), and strain EM-90 was kindly donated by Dr. Sergio Marshall. Strains CGR02 (genogroup LF89-like) and Ps12201A (genogroup EM-90 like) were isolated by Etecma (Puerto Montt, Chile) in 2013 and 2017, respectively. Strains LF-89, EM-90, and CGR02 were previously sequenced by our group, while the Ps12201A strain was sequenced in this work. The genome sequences are available at the NCBI Genome database under the accession numbers SAMN02469424, SAMN10437718, SAMN04309076, and SAMN16064558.

### Genome Sequencing and Assembly of Strain Ps12201A

Ps12201A strain was grown to exponential phase in Austral-SRS, and the total DNA was purified with the DNeasy Blood and Tissue Kit for DNA (Qiagen). The bacterial genome was sequenced using Oxford Nanopore version R9.4. DNA library was prepared from 4 μg input of DNA using the 1D Genomic DNA Ligation Sequencing Kit (SQK-LSK108) (Oxford Nanopore Technologies, Oxford, United Kingdom) in accordance with the manufacturer’s instructions. Nanopore sequencing was conducted in our lab using FLO-MIN106 (R9.4) flow cells with the software minKNOW version 1.10.11 and the script NC_48Hr_sequencing_FLO-MIN106_SQK-LSK108_plus_Basecaller for 24 h. For Illumina sequencing of Ps12201A strain, DNA library was constructed following the protocol of TruSeq DNA sample preparation (Illumina, Inc.; San Diego, CA, United States). Sequencing was performed by MrDNA Next Generation Sequencing Service Provider (Shallowater, TX, United States) on Illumina HiSeq platform in an overlapping 2 × 250 bp.

For the assembly of Ps12201A strain, Nanopore sequences were trimmed of adaptors using the software Porechop version 0.2.2. Then, they were cleaned of low-quality sequences using Nanofilt version 1.2.0 with a Phred value cutoff of *Q* > 13 and a minimum read length of 500 bp. In the case of Illumina sequences, cleaning of low-quality reads and trimming of adaptors were performed by using Trimmomatic version 0.36 with the following parameters: ILLUMINACLIP:TruSeq3-PE.fa:2:30:10, LEADING:3, TRAILING:3, SLIDINGWINDOW:4:15, and MINLEN:100. To obtain a high-quality genome assembly, the ‘‘ONT assembly and Illumina polishing pipeline’’ was used^1^. This pipeline uses Nanopore reads to build the assembly with Canu software (Koren et al., 2017), and then, these contigs are corrected using Illumina reads with Racon software^2^ and Pilon software (Walker et al., 2014). The default parameters were used with an estimated genome size of 3.2 Mb.

### Bacterial Growth and Culture Media

The strains were stored at −80°C in cryovials stocks, plated in Austral-SRS agar plates (Mikalsen et al., 2007; Vera et al., 2012), and incubated at 18°C until visible growth. The bacteria were routinely maintained by subculturing in liquid Austral-SRS medium with agitation (180 rpm) at 18°C. The *P. salmonis* culture experiments were carried out in culture broths with different nutrient composition and availabilities: (1) the nutrient-rich medium Austral-SRS; (2) the basal medium described in Cortés et al. (2017), named here CMM1; and (3) CMMAB, a new defined culture medium based on the CMM1 composition that was developed in this work to evaluate antibiotic susceptibility in agreement with the Clinical and Laboratory Standards Institute (CLSI) (Miller et al., 2014; CLSI National Committee for Clinical Laboratory Standards, 2015). All broth compositions and nutrient concentrations are detailed in Supplementary Table 1.

### Growth Curve Analysis

For growth curve assays, exponentially growing bacteria (OD_600_ = 0.3–0.7) were used as inoculum in Austral-SRS, CMM1, or CMMAB broths, and 48-well plates were inoculated to a final OD_600_ = 0.01 in 700 μl and incubated at 18°C with agitation. Every growth curve experiment was performed with two or three technical replicates and three biological replicates. Bacterial growth was evaluated periodically by measuring the optical density (OD_600_) for 7 days in an Infinite^®^ 200 PRO NanoQuant (Tecan^®^). pH measurements were conducted with an aliquot of the cultures (with or without bacteria), using pH test paper bromocresol purple pH 5.62–7.2 and pH test paper cresol red pH 7.2–8.8 (Thomas Scientific).

The effect of ampicillin in growth curves was evaluated for the LF-89 strain in 48-well plates as before but adding ampicillin to Austral-SRS and CMMAB media to a final concentration of 50 μg/ml.

Growth curve parameters were calculated based on the raw growth data using the R package Growthcurver (Sprouffske and Wagner, 2016). Growth parameters were obtained for each biological replicate, and the data were subsequently processed in Excel to compare replicates separately. GraphPad Prism version 8.0.1 for Windows (GraphPad Software, La Jolla, CA, United States) was used for graphical representation and statistical analysis of the results.

### CHSE-214 Infections and MTT Cell Viability Assay

The epithelial-like embryo cell line CHSE-214 (ECACC 91041114), derived from Chinook salmon *Oncorhynchus tshawitscha*, was obtained from the European Collection of Authenticated Cell Cultures and grown in minimum essential medium (MEM) supplemented with 5% FBS (Gibco). Infection with *P. salmonis* was carried out as described previously (Ortiz-Severín et al., 2019) with some modifications. CHSE-214 cells were seeded in T25 flasks and incubated at 20°C until reaching 80% confluency. Grown flasks were used to seed 24-well plates with 75,000 cells per well. Bacteria were grown in liquid broth (Austral-SRS or CMMAB) for 4 days and inoculated at a multiplicity of infection (MOI) of 100. After 3 days of co-incubation, gentamicin was added to a final concentration of 50 μg/ml to kill extracellular bacteria. The antibiotic was incubated for 1 h, washed three times with PBS, and replaced with fresh culture media. Infection was carried out for 14 days at 18°C. MTT cell viability assay was performed as previously described (Mosmann, 1983; Tada et al., 1986). Briefly, cultures were treated with 50 μg/ml gentamicin and PenStrep 1× solution (Gibco) for 1 h and washed with PBS before adding 300 μl of fresh MEM. Next, 30 μl of MTT solution (5 mg/ml in PBS) was added to infected and uninfected cells and plates were incubated for 5 h at 20°C protected from light. After the incubation period, supernatant from each well was carefully removed and placed in a new plate before adding 300 μl of solubilization solution (10% SDS in HCL 0.01 M) directly to the remaining cells. Cells were vigorously suspended, and the plate was incubated at room temperature for 15 min. The 300 μl of MTT supernatant solution was added to each well and mixed thoroughly before reading the absorbance at 570 and 690 nm in an Infinite^®^ 200 PRO NanoQuant (Tecan^®^) spectrophotometer. Cell viability was calculated as a percent of the control uninfected cell’s viability using the following formula: [(abs_570*nm*_sample − abs_690*nm*_sample)/abs_570*nm*_control − abs_690*nm*_ control]^∗^100.

### Prediction of Antibiotic Resistance Genes

The online interface of the Resistance Gene Identifier (RGI) version 5.2.0^3^ was used. The ARGs selection criteria were set to “Perfect,” “Strict,” and “Loose” algorithms to search for hits in the curated reference sequences in the Comprehensive Antibiotic Resistance Database (CARD) (Alcock et al., 2020) version 3.1.2. In addition, *P. salmonis* genomes were subjected to ARGs searches employing the SARG collection of hidden Markov models (HMMs) with the online version of SARGfam available in https://smile.hku.hk/SARGs which uses HMMER3 in hmmscan mode (Yin et al., 2018). Hits with an e-value cutoff of 1 × 10^–5^ were annotated as ARG. Finally, we searched for ARGs using AMRFinder software (Feldgarden et al., 2019) as standalone version^4^ and the parameters were set to *i* = 0 and *c* = 0.

Afterward, all results were manually curated, filtered by sequence identity over 35% and coverage over 50%, and categorized by hand using the CARD categories available^5^ without incorporating duplicated results. The bar graphs and bubble chart were created using GraphPad Prism version 8.0.1 for Windows; this program was used for graphical representation and statistical analysis of the results. Figures were composed using Adobe Illustrator CS6.

### Antibiotic Susceptibility Testing

Minimal inhibitory concentration (MIC) values were determined for four *P. salmonis* strains using two-fold dilutions of the antibiotics tetracycline (10 μg/ml), oxytetracycline (10 μg/ml), flumequine (40 μg/ml), oxolinic acid (10 μg/ml), erythromycin (100 μg/ml), chloramphenicol (20 μg/ml), florfenicol (20 μg/ml), polymyxin B (100 μg/ml), vancomycin (10 mg/ml), ampicillin (800–1000 μg/ml), penicillin G (800 μg/ml), ceftazidime (10 mg/ml), streptomycin (400 μg/ml), spectinomycin (100 μg/ml), trimethoprim (20 mg/ml), and rifampicin (100 μg/ml). Antibiotic stocks were prepared and stored according to the CLSI recommendations (Miller et al., 2014). The antibiotic susceptibility testing was performed according to the CLSI guidelines (Miller et al., 2014), using the Austral-SRS nutrient broth and the defined medium CMMAB in 96-well round-bottom plates. Freshly prepared two-fold dilutions were used in each microplate inoculated with exponentially growing cultures at a final OD_600_ = 0.01. The plates were sealed and incubated statically at 18°C for 7 days (strains LF-89, CRG02) or 10 days (strains EM-90 y Ps12201A). The MIC value was defined as the lowest concentration of the antibiotic that inhibited at least 80% of normal growth, considering the normal growth as a bacterial precipitate with a diameter of >2 mm. Each plate contained three replicates per condition, and every experiment was repeated at least five times.

### Clover-Leaf Test

β-Lactamase production was evaluated in *P. salmonis* as described before (Mikalsen et al., 2007). Briefly, *E. coli* DH5α was used as the indicator strain and *P. salmonis* LF-89 as the test strain. Overnight cultures of bacteria grown in Austral-SRS broth were used as inoculum for the Austral-SRS and CMMAB agar plates. *P. salmonis* was streaked over the *E. coli* lawn, and two disks containing 100 or 500 mg of ampicillin were placed over the *P. salmonis* lines. After 10 days of incubation at 18°C, plates were photographed, and the inhibition zones were evaluated.

### Bacterial RNA Purification and Transcripts Quantification

Total RNA was purified from *P. salmonis* cultures grown in Austral-SRS, CMMAB, or CMMAB supplemented with 50 μg/ml ampicillin and grown to mid-log phase (OD_600_ ∼ 0.6). *P. salmonis* cells were collected by centrifugation at 8,000 × *g* for 5 min, washed with sterile PBS, and suspended in TRIzol (Thermo Fisher Scientific). Cells were disaggregated and homogenized with a 27G syringe, and RNA was purified following the manufacturer’s instructions. RNA was suspended in Ambion^®^ RNAsecure^TM^ (Invitrogen), treated with RQ1 RNase-Free DNase I (Promega) according to standard protocols, and visualized in an RNA denaturing agarose gel. The purified RNA was quantified using a Qubit^TM^ RNA HS Assay kit (Thermo Fischer Scientific), and 2 μg of RNA was used to synthesize cDNA with the M-MLV Reverse Transcriptase using random primers (Promega). Transcripts were quantified using a Takyon qPCR Kit (Eurogentec) with specific primers designed for the predicted *P. salmonis* LF-89 ARGs (Supplementary Table 5). Real-time quantitative PCR (qPCR) was performed in an AriaMx 1.0 system (Agilent). The geometric median of the housekeeping genes *glyA* and *pykA* was calculated for each sample and used to calculate the relative expression levels of the *P. salmonis* genes using the method described by Pfaffl (2001).

### RNA Sequencing and Analysis

Three independent cultures of *P. salmonis* LF-89 grown in Austral-SRS broth for 2 days were used to inoculate 5-ml cultures of Austral-SRS, CMMAB, and CMMAB supplemented with 50 μg/ml ampicillin, each in triplicate. When cultures reached OD_600_ ∼ 0.6, 2 ml of each culture was collected and centrifuged at 8,000 × *g* for 5 min. As before, bacteria were disaggregated and homogenized with a 27G syringe, and RNA was purified following the manufacturer’s instructions using RNeasy kit (Qiagen), suspended in RNA secure (Ambion), treated with RNase-Free DNase I as described before, and frozen at −80°C until use. Water-diluted samples were quantified with Qubit and RNA integrity was analyzed by TapeStation (Agilent). Approximately 20 μg of RNA of each sample was sent to Novogene (United States) for library preparation and Illumina sequencing. Nine libraries (three for each condition) were prepared and sequenced by Novogen Bioinformatics Technology Co., Ltd. (Beijing, China). The libraries were generated using TruSeq Stranded Total RNA Library Prep (Illumina), and RiboZero Magnetic Kit (Illumina) to remove rRNAs. The sizes of inserts selected for amplification were 250–300 bp. The libraries were sequenced in an Illumina Novaseq platform using 150-bp size paired-end reads. A total of 30.5G raw RNA-seq reads per library were obtained. RNA-seq has been submitted to NCBI, accession number PRJNA744511.

Read quality of the three replicates for every medium (nine samples in total) was assessed with the FASTQC software v0.11.5 (Andrews, 2010), and a proper primer quality trimming was performed using BBDUK from the BBTOOLS suit (Bushnell, 2014). Forward and reverse reads were mapped to the latest *P. salmonis* LF-89 assembly (NCBI accession GCF_000300295.4) using BWA v0.7.17 (Li and Durbin, 2009) and the resulting files were converted to a sorted-bam format with the use of Samtools v1.7 (Li et al., 2009). Over 3,000 genes of LF-89 strain were counted using the HTSeq-count script v 0.6.1 from the HTSeq framework (Anders et al., 2015) along with the previous sorted-bam output.

Samples were compared by PCA using their global gene expression patterns with the Prcomp function of the Stats package v3.6.3 in R v3.6.3 (R Core Team, 2015). For visualizations, gene expression levels were normalized using the between-sample normalization method Trimmed Mean of M-values method (TMM), in addition to the inter-sample normalization method Counts per million mapped reads (CPM). Finally, expression levels were transformed with the pseudo-log_2_ method.

Gene-counted files for every sample were then analyzed with an in-house R script, using the DESeq2 library for differential expression analysis (Love et al., 2014). Considering sample S (*P. salmonis* grown in Austral-SRS), sample C (*P. salmonis* grown in CMMAB), and sample A (*P. salmonis* grown in CMMAB with ampicillin), the differential expression was tested between A/S and C/S with S as reference, and A/C with C as reference. Only genes detected (>0 counts) in at least two of three biological replicates were analyzed. The complete list with gene counts and annotation is shown in Supplementary Table 3.

Annotated genes were classified according to the predicted functions in the Clusters of Orthologous Groups (COGs) database (Tatusov, 2000). GO enrichment analysis was performed using the ClueGO tool (Bindea et al., 2009) in pairwise comparisons (A/S and C/S comparisons against the reference genome). A *p*-value < 0.05 and kappa coefficient of 0.4 were considered as threshold values, and only unduplicated GO terms from biological processes with *p*-value < 0.01 were incorporated in the graphs. The complete list of enriched GO terms is found in Supplementary Table 4.

### Metabolic Pathway Analysis

A preliminary analysis of metabolic pathways linked to differentially expressed genes was carried out by mapping transcriptomic data to the genome-scale model of *P. salmonis* LF-89 (Cortés et al., 2017). Subsystem data were complemented with manually curated pathway information on Metacyc (Karp et al., 2018) to obtain a list of metabolic pathways of interest. These pathways were subsequently represented on a simplified form using Escher (King et al., 2015) assigning a fold-change value for each reaction according to their associated genes. For reactions associated with multiple transcripts, computation of fold-change values was performed based on their GPR (gene protein reaction) logical rule. If an OR connector was used, then the associated value corresponds to the maximum value of the fold changes. On the other hand, if an AND connector was used, then the associated fold change for this reaction was its minimum. The complete list of genes mapped to the metabolic pathways and their expression levels can be found in Supplementary Table 4.

## Data Availability Statement

The datasets presented in this study can be found in online repositories. The names of the repository/repositories and accession number(s) can be found below: https://www.ncbi.nlm.nih.gov/bioproject/, PRJNA744511.

## Author Contributions

JO-S, VC, and AM conceived and designed the study. JO-S developed the defined medium and performed bacterial growth assays. JO-S and CS performed ARG predictions and antibiotic susceptibility testing. JO-S carried out RNA extraction, interpreted the RNA-seq results, and prepared the figures. NJ and MC carried out the analysis of metabolic pathways and prepared the figures. JM and RP performed bioinformatics and statistical analyses. AM and VC contributed with valuable feedback and funding acquisition. JO-S and VC wrote this article with input from all other authors. All authors reviewed the manuscript.

## Conflict of Interest

The authors declare that the research was conducted in the absence of any commercial or financial relationships that could be construed as a potential conflict of interest.

## Publisher’s Note

All claims expressed in this article are solely those of the authors and do not necessarily represent those of their affiliated organizations, or those of the publisher, the editors and the reviewers. Any product that may be evaluated in this article, or claim that may be made by its manufacturer, is not guaranteed or endorsed by the publisher.

## Footnotes

1
https://github.com/nanoporetech/ont-assembly-polish

2
https://github.com/isovic/racon

3
https://card.mcmaster.ca/analyze/rgi

4
https://github.com/ncbi/amr/wiki/AMRFinder-database

5
https://card.mcmaster.ca/
