# Supplementary figures and images for: Nutrient Scarcity in a New Defined Medium Reveals Metabolic Resistance to Antibiotics in the Fish Pathogen Piscirickettsia salmonis
Source: Front Microbiol. 2021 Oct 11;12:734239. doi: 10.3389/fmicb.2021.734239 (PMC8542936; doi:10.3389/fmicb.2021.734239)

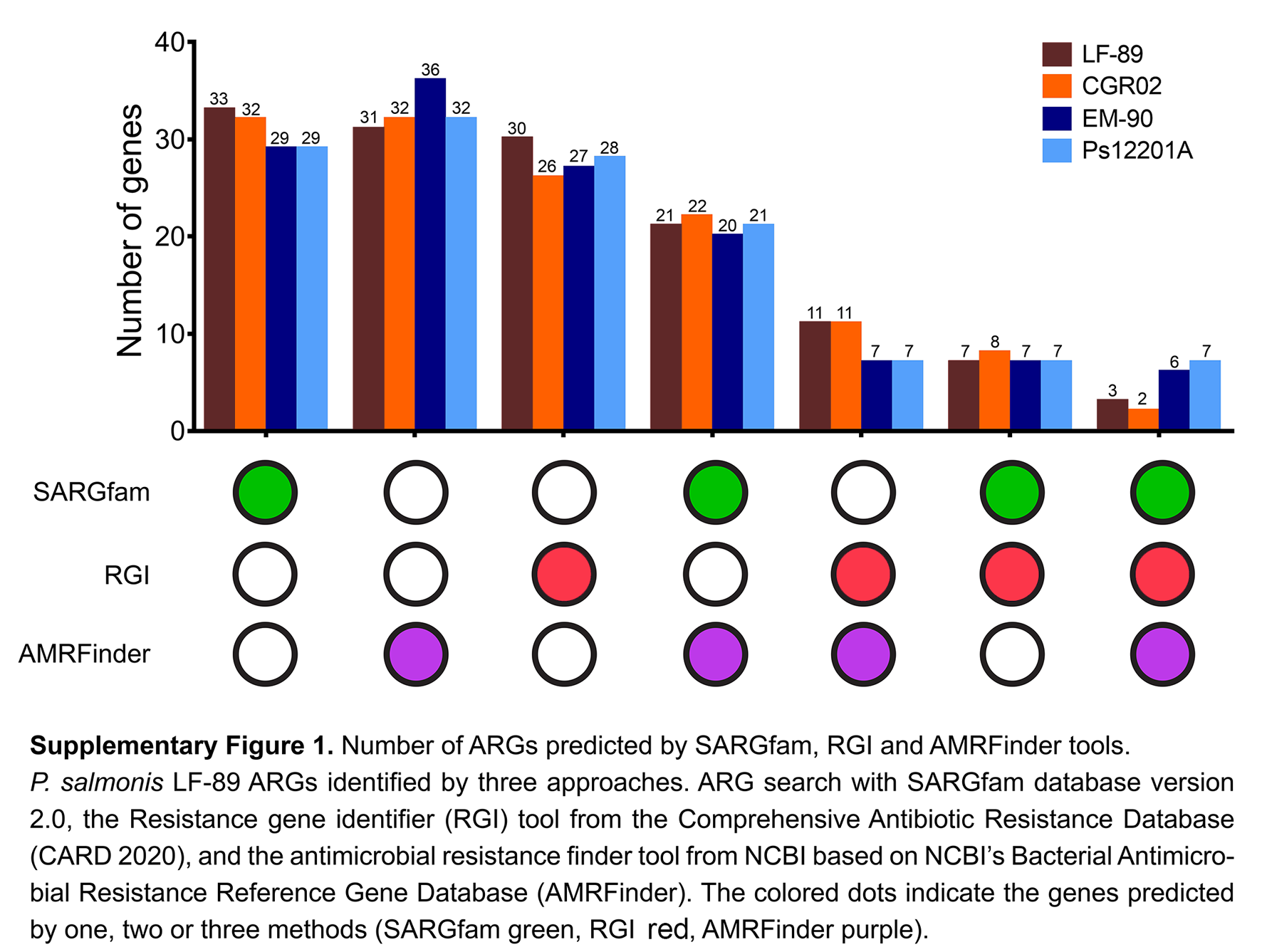

Supplement: Supplementary file 1 [file Image_1.TIF]

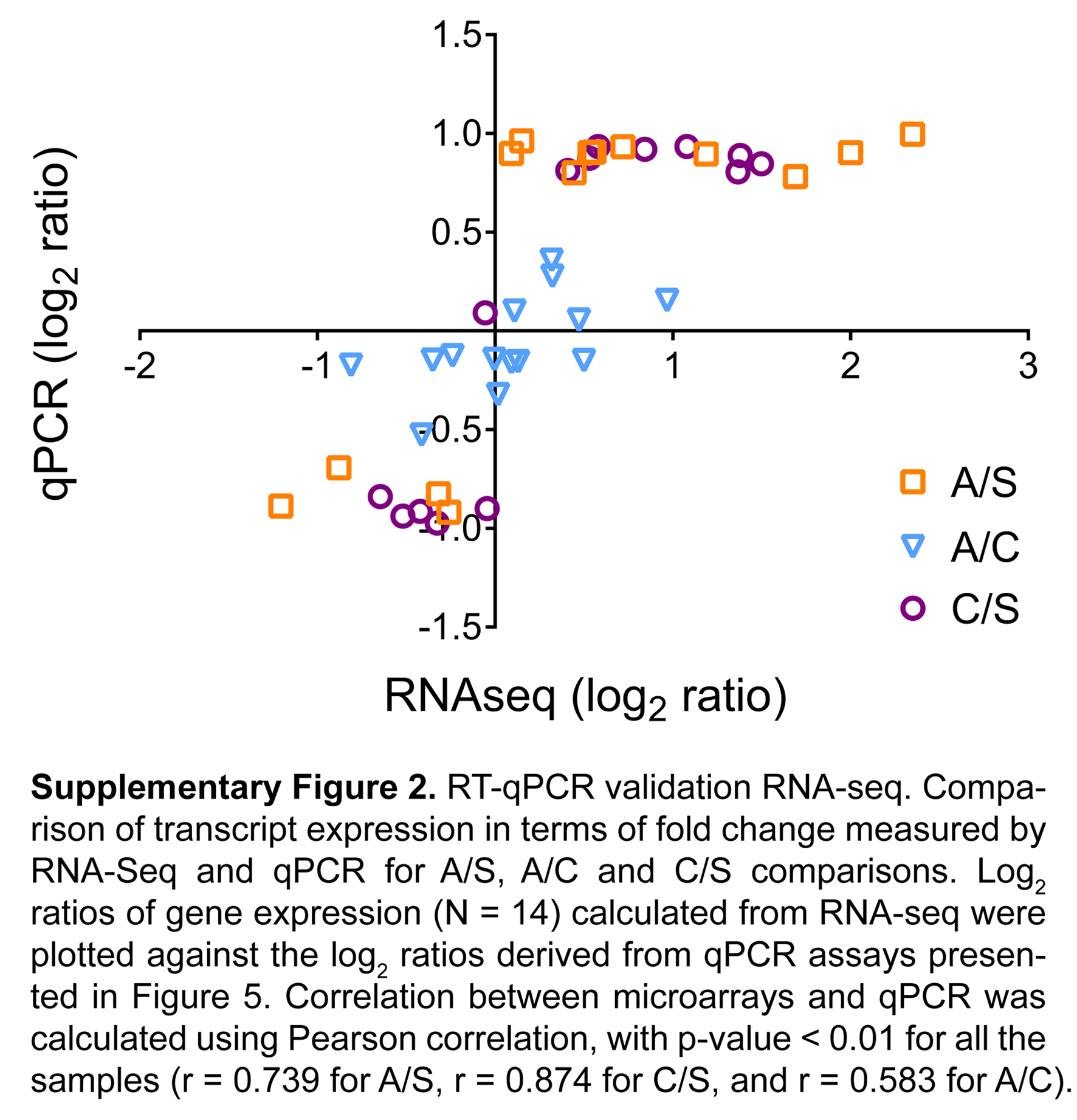

Supplement: Supplementary file 2 [file Image_2.TIF]

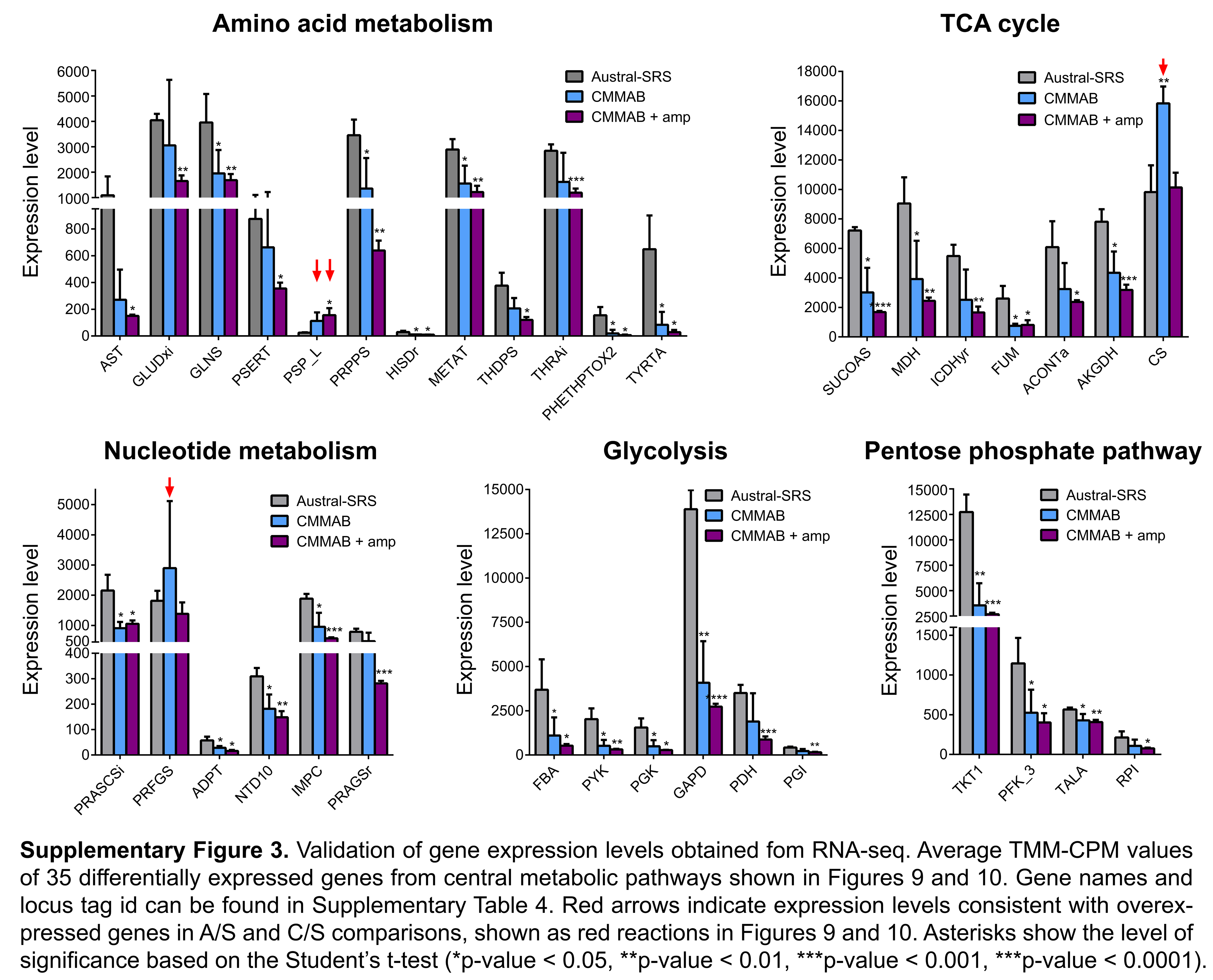

Supplement: Supplementary file 3 [file Image_3.TIF]
