# Supplementary material for: Nutrient Scarcity in a New Defined Medium Reveals Metabolic Resistance to Antibiotics in the Fish Pathogen Piscirickettsia salmonis
Source: Front Microbiol. 2021 Oct 11;12:734239. doi: 10.3389/fmicb.2021.734239 (PMC8542936; doi:10.3389/fmicb.2021.734239)
Supplement: Supplementary file 5 [file Table_2.pdf]

Supplementary Table 2. Resistance mechanism classification of predicted ARGs. The number of genes classified at each resistance mechanism is shown for *P. salmonis* strains LF-89, CGR02, EM-90 and Ps12201A. Note that some ARGs could be classified in more than one resistance mechanism.

	**LF-89**			**CGR02**			**EM-90**			**Ps12201A**		
**Resistance mechanism**	**SARG-fam**	**RGI**	**AMR-Finder**	**SARG-fam**	**RGI**	**AMR-Finder**	**SARG-fam**	**RGI**	**AMR-Finder**	**SARG-fam**	**RGI**	**AMR-Finder**
antibiotic efflux	33	23	34	33	22	34	32	21	30	38	24	29
antibiotic inactivation	1	3	2	1	2	2	0	2	2	1	2	2
antibiotic target alteration	33	21	18	33	20	18	31	22	20	26	21	21
antibiotic target protection	0	4	8	0	3	9	0	3	14	0	3	12
antibiotic target replacement	0	2	4	0	2	4	0	2	3	0	2	3
