# Supplementary material for: Nutrient Scarcity in a New Defined Medium Reveals Metabolic Resistance to Antibiotics in the Fish Pathogen Piscirickettsia salmonis
Source: Front Microbiol. 2021 Oct 11;12:734239. doi: 10.3389/fmicb.2021.734239 (PMC8542936; doi:10.3389/fmicb.2021.734239)
Supplement: Supplementary file 8 [file Table_5.pdf]

Supplementary Table 5. Primers used in this study.

Locus tag	Predicted ARG name	Primer forward	Primer reverse
PSLF89_RS22030	*ABC_transporter*	AGCACGCGCCTATCTAAAAA	GTGAAGGGCGATGAGTGATT
PSLF89_RS22225	*tolC*	ACGATCAAGACGCAGGAAAC	TGAAGCTGCGCGTGTATAAG
PSLF89_RS23190	*evgS*	ACGCACAGAGCAACTCAATG	GAGGCGAGCTTTTCTGATTG
PSLF89_RS23435	*emrD/fexA*	CGCTGCTTGGCCTTATTATC	AGGCAGGATAATGGCAGTTG
PSLF89_RS23680	*bcr*	AGCCACCCTTATCAAACACG	ACGCTAAGGCGTTTAGAGCA
PSLF89_RS25885	*ABC_transporter /msbA*	TGGTGTCATGCTTTCAGGAG	ATTATCCAGTGCGGAGGTTG
PSLF89_RS27025	*mecD2*	AGCAACCCCGTTACAGATTG	TCTTTGATCACCCGATAGCC
PSLF89_RS28565	*cpxR*	AACTGCACGTGGTGAAGAAG	TCTAAGCACCGCCCTTAAAC
PSLF89_RS29285	*golS*	CATGAGGTGCATGAAGTTGC	ACCAAAGCGTGTAACCCATC
PSLF89_RS31110	*marR*	CAGGACTCACGCAAGTTCAA	GGGGGTAAGGTAGAGGCAAC
PSLF89_RS32405	*PBP1B*	GCGTCAGGCCACTAAAATTG	GTGTTTGAGCATCGTTGAGC
PSLF89_RS33080	*PBP1A*	CTAAGCGCACCCAAGAACTC	GACGGTGGTGACAACATCTG
PSLF89_RS33730	*mecD1*	TGCGCTATCCTTTGAGTGTG	TGAACCATAGCACGATGTCC
PSLF89_RS36830	*nmcR*	TTGACAGAAGCAGGTCAAGC	GCAGCCAATCGTTAAGGTTC
